# Serum Uric Acid and Kidney Disease Measures Independently Predict Cardiovascular and Total Mortality: The Uric Acid Right for Heart Health (URRAH) Project

**DOI:** 10.3389/fcvm.2021.713652

**Published:** 2021-09-27

**Authors:** Elisa Russo, Francesca Viazzi, Roberto Pontremoli, Carlo M. Barbagallo, Michele Bombelli, Edoardo Casiglia, Arrigo F. G. Cicero, Massimo Cirillo, Pietro Cirillo, Giovambattista Desideri, Lanfranco D'Elia, Raffaella Dell'Oro, Claudio Ferri, Ferruccio Galletti, Loreto Gesualdo, Cristina Giannattasio, Guido Iaccarino, Giovanna Leoncini, Francesca Mallamaci, Alessandro Maloberti, Stefano Masi, Alessandro Mengozzi, Alberto Mazza, Maria L. Muiesan, Pietro Nazzaro, Paolo Palatini, Gianfranco Parati, Marcello Rattazzi, Giulia Rivasi, Massimo Salvetti, Valérie Tikhonoff, Giuliano Tocci, Fosca A. L. Quarti Trevano, Andrea Ungar, Paolo Verdecchia, Agostino Virdis, Massimo Volpe, Guido Grassi, Claudio Borghi

**Affiliations:** ^1^Department of Internal Medicine, University of Genoa and IRCCS Ospdedale Policlinico San Martino, Genova, Italy; ^2^Biomedical Department of Internal Medicine and Specialistics, University of Palermo, Palermo, Italy; ^3^Clinica Medica, Department of Medicine and Surgery, University of Milano-Bicocca, Monza, Italy; ^4^Studium Patavinum, Department of Medicine, University of Padua, Padua, Italy; ^5^Department of Medical and Surgical Science, Alma Mater Studiorum University of Bologna, Bologna, Italy; ^6^Department of Public Health, Federico II University of Naples Medical School, Naples, Italy; ^7^Department of Emergency and Organ Transplantation-Nephrology, Dialysis and Transplantation Unit, Aldo Moro University of Bari, Bari, Italy; ^8^Department of Life, Health and Environmental Sciences, University of L'Aquila, L'Aquila, Italy; ^9^Department of Clinical Medicine and Surgery, Federico II University of Naples Medical School, Naples, Italy; ^10^Cardiology IV, A. De Gasperis Department, School of Medicine and Sugery, Niguarda Ca' Granda Hospital, Milano-Bicocca University, Milan, Italy; ^11^Department of Advanced Biomedical Sciences, Federico II University of Naples Medical School, Naples, Italy; ^12^CNR-IFC, Clinical Epidemiology of Renal Diseases and Hypertension, Reggio Cal Unit, Reggio Calabria, Italy; ^13^Department of Clinical and Experimental Medicine, University of Pisa, Pisa, Italy; ^14^Department of Internal Medicine, Hypertension Unit, General Hospital, Rovigo, Italy; ^15^Department of Clinical and Experimental Sciences, University of Brescia, Brescia, Italy; ^16^Department of Medical Basic Sciences, Neurosciences and Sense Organs, University of Bari Medical School, Bari, Italy; ^17^Department of Medicine and Surgery, University of Milano-Bicocca, Milan, Italy; ^18^Department of Cardiology, San Luca Hospital, Istituto Auxologico Italiano, IRCCS, Milan, Italy; ^19^Department of Medicine, Ca' Foncello University Hospital, University of Padova, Treviso, Italy; ^20^Department of Geriatric and Intensive Care Medicine, Careggi Hospital, University of Florence, Florence, Italy; ^21^Department of Medicine, University of Padua, Padua, Italy; ^22^Hypertension Unit, Division of Cardiology, Department of Clinical and Molecular Medicine, Faculty of Medicine and Psychology, Sant'Andrea Hospital, University of Rome Sapienza, Rome, Italy; ^23^Hospital S. Maria della Misericordia, Perugia, Italy

**Keywords:** hyperuricemia, eGFR, albuminuria, cardiovascular mortality, all-cause mortality

## Abstract

**Background:** Serum uric acid predicts the onset and progression of kidney disease, and the occurrence of cardiovascular and all-cause mortality. Nevertheless, it is unclear which is the appropriate definition of hyperuricemia in presence of chronic kidney disease (CKD). Our goal was to study the independent impact of uric acid and CKD on mortality.

**Methods:** We retrospectively investigated 21,963 patients from the URRAH study database. Hyperuricemia was defined on the basis of outcome specific cut-offs separately identified by ROC curves according to eGFR strata. The primary endpoints were cardiovascular and all-cause mortality.

**Results:** After a mean follow-up of 9.8 year, there were 1,582 (7.20%) cardiovascular events and 3,130 (14.25%) deaths for all causes. The incidence of cardiovascular and all-cause mortality increased in parallel with reduction of eGFR strata and with progressively higher uric acid quartiles. During 215,618 person-years of follow-up, the incidence rate for cardiovascular mortality, stratified based on eGFR (>90, between 60 and 90 and <60 ml/min) was significantly higher in patients with hyperuricemia and albuminuria (3.8, 22.1 and 19.1, respectively) as compared to those with only one risk factor or none (0.4, 2.8 and 3.1, respectively). Serum uric acid and eGFR significantly interact in determining cardiovascular and all-cause mortality. For each SUA increase of 1 mg/dl the risk for mortality increased by 10% even after adjustment for potential confounding factors included eGFR and the presence of albuminuria.

**Conclusions:** hyperuricemia is a risk factor for cardiovascular and all-cause mortality additively to eGFR strata and albuminuria, in patients at cardiovascular risk.

## Introduction

Serum uric acid (SUA) has been shown to predict the occurrence of cardiovascular (CVM) and all-cause mortality (ACM) (1) as well as the onset and progression of chronic kidney disease (CKD) (2).

Chronic kidney disease is also a well-known risk multiplier in itself. Both reduced GFR and albuminuria independently entail an increased risk of mortality, especially from CV causes (3). Many risk factors have been identified for predicting CVM in patients with kidney impairment, such as smoking, diabetes, blood pressure (BP), inflammation and hypertension-mediated organ damage (4–6). Several (7–9) but not all (10, 11) studies have suggested that hyperuricemia (HU) may be an additional potential risk factor for CVM in individuals with CKD.

CKD and HU often coexist as glomerular filtration rate (GFR) reduction is one of the main determinants of UA increase. Thus, it is difficult to sort out the independent role of each one of these conditions.

We have previously reported on the specific threshold of SUA associated to a significant increase in CVM and ACM (12) in a large, observational cohort of patients at cardiovascular risk. However, it is at present unclear which is the proper definition of HU in presence of chronic kidney disease and whether HU and renal impairment are independent risk factor for CV and all-cause mortality.

Our aim was to investigate the relationship between SUA and CKD components in causing mortality.

## Methods

### Population

The Uric Acid Right for Heart Health (URRAH) project is based on a multicenter retrospective, observational cohort study, which involves data collected on a regional community basis from all the territory of Italy under the patronage of the Italian Society of Hypertension. Participant centers who collected the data included in the general database are listed under Acknowledgements. The study protocol has been previously extensively described (12). A nationwide Italian database was constructed by merging data from several cohorts recruited within the Italian Centers of Hypertension and distributed in almost all the Italian regions. These observational cohort studies included Caucasian individuals, 18–95 years old having SUA measurement and complete information about several variables including demographics, metabolic parameters, smoking habit, systolic and diastolic BP, kidney function, concomitant treatments and outcomes.

The URRAH was performed according to the Declaration of Helsinki for Human Research (41st World Medical Assembly, 1990). The processing of the patients' personal data collected in this study comply with the European Directive on the Privacy of Data. All data to be collected, stored and processed are anonymized, and all study-related documents are retained in a secure location. No personal information is stored on local personal computers. Approval was sought from the Ethical Committee of the coordinating center at the Division of Internal Medicine of the University of Bologna (no. 77/2018/Oss/AOUBo). Informed consent was obtained from all individuals at recruitment.

### Data Collection

Hypertension was defined according to ESH-ESC guidelines as a BP at least 140/90 mmHg or by the presence of antihypertensive treatment. Systolic and diastolic BP was measured twice, in a quiet room, after 5 min resting and with the participant in sitting position. The second measure was used for all analyses.

Data on the patients (*n* 8,607; 4,697 men, 3,910 women) for whom the SUA was not available or the estimation of eGFR was not possible or follow-up data were not complete were excluded from analysis. Data obtained from the remaining 21,963 patients (10,981 men, 11,072 women) form the basis of the present study.

Kidney function was assessed by serum creatinine and urinary albumin excretion measurements. GFR was estimated for each patient using a standardized serum creatinine assay and the Chronic Kidney Disease Epidemiology Collaboration formula (13). Urine samples for albumin excretion measurements were collected before each study visit (usually within 1 week). Abnormal urinary albumin excretion was diagnosed if urinary albumin concentration was >30 mg/L, or if urinary albumin excretion rate was >20 μg/min, or if urinary albumin-to-creatinine ratio (ACR) was >3.4 mg/mmol or >30 mg/g in both genders. Albuminuria indicates patients with either micro or macroalbuminuria. Chronic kidney disease was defined for estimated glomerular filtration rate (eGFR) values eGFR <60 ml/min per 1.73 m^2^ and/or albuminuria.

### Outcomes

The main analysis was aimed at evaluating the association between SUA and the development of outcomes during the follow-up study period. The following hard endpoints were evaluated at the end of the follow-up: CVM as a composite of fatal events due to acute myocardial infarction, sudden cardiac death, heart failure, or stroke and ACM. Information about death was obtained from hospital records or death certificates.

### Statistical Analyses

Baseline clinical and demographical patient's characteristics were reported overall and per eGFR strata as mean and SD for continuous variables normally distributed and as median (interquartile ranges) for skewed variables. Logarithmically transformed values of skewed variables were used for the statistical analysis. Comparisons between groups were made by analysis of variance. Comparisons of proportions among groups were made using the χ^2^ test or Fisher's exact test when appropriate. Missing values, when present, were below 5%.

Given the known difference in SUA levels along with eGFR strata, we tested SUA for an interaction with eGFR in its association with the two clinical outcomes.

Furthermore, three CKD-specific cutoff points were identified, and their prognostic value for CVM and ACM was tested. To this purpose, the survival receiver operating characteristic curve was implemented using Kaplan-Meier estimates and was used to search for prognostic cut-off of SUA that optimized the combination of sensitivity (true-positive) and 1-specificity (false-positive) for CVM and ACM by the presence of CKD (14). Youden's index (15) defined for all points of ROC curves was used as a criterion for selecting the optimum cut-off.

Time to event analyses were performed using: (i) Kaplan-Meier method for survival curves estimation and log-rank test to compare them; (ii) univariate and multivariate Cox regression models: risk was reported as hazard ratios (HR) along with their 95% confidence intervals (CI). Covariates included all available clinical variables with biological plausibility. Time variable was defined as the interval time between baseline date and the date of endpoint occurrence or the last available follow-up.

Power analysis showed that the number of individuals in the database (*n* = 21,963) represented a sample largely sufficient to avoid b error also after stratification by CKD.

Statistical calculations were performed by STATA package, version 14.2 (StataCorp, 4905 Lakeway Drive, College Station, Texas 77845 USA). The null hypothesis was rejected for values of *P* < 0.05.

## Results

### Baseline Characteristics of URRAH Study Cohort on the Basis of eGFR Strata

Baseline characteristics are presented in Table 1 for the overall cohort and by eGFR levels. The study cohort was composed of 21,963 patients aged 58 ± 15 years, 54% males. At enrollment, 5271 participants 24% were current or former smokers, 68 (*n* = 14935) and 7% (*n* = 1537) had a positive history for hypertension and diabetes, respectively. Office systolic BP was 144 ± 23 mm Hg, and diastolic BP was 85 ± 13 mm Hg. Mean SUA was 5.03 mg/dl and mean eGFR was 81.5 ± 20.0 ml/min/1.73 m2. When we analyzed the baseline clinical characteristics across eGFR stage (>90, 90–60 and <60 ml/min/1.73 m^2^) we found that patients with preserved eGFR (i.e., >90 ml/min/1.73 m^2^) were younger, more frequently males, without a positive history of hypertension and diabetes and with lower BMI, BP levels, fasting glucose and a more favorable lipid profile as compared to those with impaired renal function. As for mean SUA levels, HU, gout and use of Allopurinol they were all lower in patients with eGFR above 90 ml/ min (Table 1).

**Table 1:** Baseline clinical characteristics of study patients on the basis of eGFR strata.

**Baseline characteristics**	**All**	**eGFR ≥ 90 ml/min per 1.73 m^**2**^**	**eGFR 60–90 ml/min per 1.73 m^**2**^**	**eGFR <60 ml/min per 1.73 m^**2**^**	***P*-value**
*N*	21,963	7,354	11,262	3,167	
Age, years	58 ± 15	48 ± 13	61 ± 13	68 ± 12	<0.001
Male sex, %	49.6	64	48	19	<0.001
Body mass index, kg/m^2^	27 ± 4	26 ± 4	27 ± 4	27 ± 4	<0.001
Smokers, %	23.5	27	22	21	<0.001
Hypertension, %	64.2	51.8	68	78	<0.001
Systolic blood pressure, mmHg	144 ± 23	137 ± 22	146 ± 23	152 ± 23	<0.001
Diastolic blood pressure, mmHg	85 ± 13	84 ± 13	86 ± 12	86 ± 13	<0.001
Heart rate, bpm	72 ± 12	72 ± 12	72 ± 2	71 ± 12	<0.001
Creatinine, mg/dl	0.93 ± 0.26	0.78 ± 0.12	0.94 ± 0.13	1.26 ± 0.46	<0.001
eGFR, ml/min per 1.73 m^2^	81 ± 20	103 ± 10	76 ± 8	50 ± 9	<0.001
eGFR <60 ml/min per 1.73 m^2^, %	14.4	0	0	100	<0.001
Normo-micro-macroalbuminuria, %	89.8 - 9.3 - 0.9	89.6 - 9.4 - 0.9	90.5 - 8.7 - 0.7	88.1 - 10.6 - 1.3	0.314
Serum uric acid, mg/dl	5.02 ± 1.42	4.55 ± 1.30	5.10 ± 1.35	5.88 ± 1.48	<0.001
Hyperuricemia according to URRAH cut-off for ACM, %	55	41	59	77	<0.001
Hyperuricemia according to URRAH cut-off for CVM, %	30	19	32	53	<0.001
Gout, %	1.1	0.5	1.2	2	<0.001
Allopurinol use, %	1.5	0.8	1.1	4.4	<0.001
Hemoglobin, g/dl	14.4 ± 1.4	14.2 ± 1.4	14.5 ± 1.3	14.3 ± 1.6	<0.001
Glucose, mg/dl	99 ± 25	94 ± 22	100 ± 24	106 ± 32	<0.001
Diabetes, %	11	7	12	17	<0.001
Cholesterol, mg/dl	213 ± 39	207 ± 40	216 ± 39	212 ± 40	<0.001
HDL-cholesterol, mg/dl	55 ± 19	55 ± 18	55 ± 18	54 ± 21	0.005
LDL-cholesterol, mg/dl	133 ± 38	129 ± 37	136 ± 37	131 ± 39	<0.001
Triglycerides, mg/dl	129 ± 79	117 ± 79	133 ± 78	143 ± 78	<0.001
ACE inhibitors, %	15	11	15	25	<0.001
Angiotensin II receptor blockers, %	11	9	9	17	<0.001
Calcium channel blockers,%	8	6	9	14	<0.001
Beta blockers, %	9	9	8	12	<0.001
Diuretics, %	16	13	16	25	<0.001
Statins, %	5	4	5	10	<0.001

*Data presented as mean ± standard deviation or percentage. eGFR, estimated glomerular filtration rate; ACM, all-cause mortality; CVM, cardiovascular mortality; HDL, high-density lipoprotein; LDL, low-density lipoprotein; ACE, angiotensin converting enzyme*.

In the subgroup of 5,170 (23.5%) patients with available data about albumin excretion at baseline, we found a prevalence of micro- and macro-albuminuria of 9.3 and 0.9%, respectively. The characteristics of these patients stratified by eGFR stage and albuminuria are depicted in **Suppplementary** Table 1. Patients with lower eGFR and albuminuria were more likely aged and in the average with an unfavorable CV profile coupled to higher SUA levels, proportion of gout and allopurinol use as compared with patients with higher eGFR and normoalbuminuria.

### Cardiovascular and All-Cause Mortality

In the overall study population, median follow-up was 9.8 years. During 215,618 person-years of follow-up, 1,063 patients developed non-fatal cardiovascular events (4.8 per 1,000 person-years), 1,582 patients died due to cardiovascular disease (7.3 per 1,000 person-years) and 3,130 patients died from all causes (14.5 per 1,000 person-years).

The 1,582 patients dead for CV and the 3,130 dead for ACM (Supplementary Table 2) were older, with a positive history of hypertension, diabetes and gout as compared to survived patients. Moreover, mortality was associated with higher BP levels, and fasting blood sugar, unfavorable lipid profile, lower eGFR, and higher prevalence of albuminuria. While patients who died for CV disease were more likely to be females, this was not the case for ACM. Patients dead for CVM and ACM showed significantly higher SUA levels (5.96 ± 1.51 vs. 4.90 ± 1.41, and 5.42 ± 1.49 vs. 4.96 ± 1.40 mg/dl, respectively) and were more likely to have HU and gout (Supplementary Table 2).

**Figure 1:**
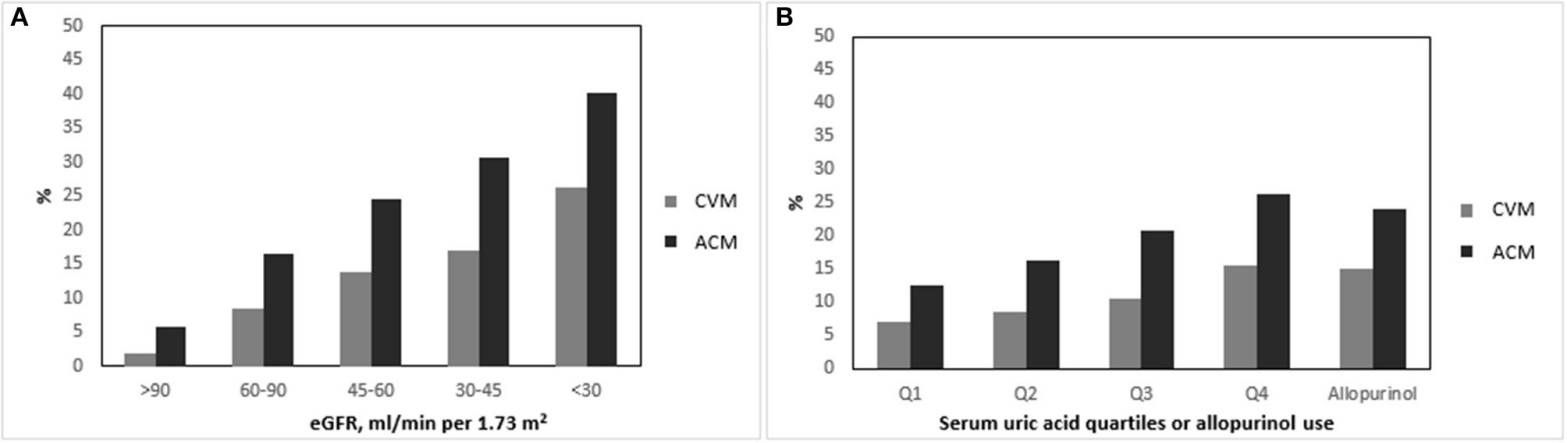
Incidence of cardiovascular and all-cause mortality on the basis of glomerular filtration rate strata **(A)**, serum uric acid quartiles and allopurinol use. **(B)** Serum uric acid quartiles defined as: Q1, SUA ≤ 3.9 mg/dl; Q2, SUA 3.9–4.8 mg/dl; Q3, SUA 4.9–5.8 mg/dl; Q4, SUA ≥ 5.9 mg/dl. CVM, cardiovascular mortality; ACM, all-cause mortality.

To better understand the relationship between HU, renal function and outcomes, we analyzed the incidence of CV an ACM on the basis of CKD strata and SUA quartiles (Figure 1). The proportion of patients who reached CVM and ACM increased in a linear fashion with decreasing of eGFR strata (<30, 30–45, 45–60, 60–90 and >90 ml/min/1.73 m^2^) and with the increasing of SUA quartiles. Patients treated with Allopurinol showed mean SUA levels higher than non-treated patients (5.86 ± 1.76 vs. 4.99 ± 1.43 mg/dl, respectively; *p* < 0.0001) and developed CVM and ACM with an incidence of 15 and 25%, respectively.

### Interaction Between Uric Acid and eGFR as Outcomes Predictors

A Cox regression interaction analysis using the two mortality outcomes assessed the interaction between SUA levels and eGFR in determining cardiovascular and all-cause mortality (*p* < 0.0001, data not shown).

To investigate the specific predictive role of serum uric acid for CVM and ACM according to eGFR strata, we performed univariate and multivariate Cox survival analysis splitting the cohort on the basis of three eGFR strata (>90, 60–90 and <60 ml/min/m^2^). Table 2 shows as the UA adjusted hazard ratios decrease with decreasing of eGFR strata both for cardiovascular (1.21 CI 95% [1.08–1.36], *p* = 0.0014; 1.13 CI 95% [1.08–1.19], *p* < 0.0001; 1.05 CI 95% [0.99–1.12], *p* = 0.1189, respectively) and all-cause mortality (1.15 CI 95% [1.07–1.23], *p* = 0.0002; 1.08 CI 95% [1.07–1.15], *p* < 0.0001; 1.10 CI 95% [1.00–1.10], *p* = 0.0502; respectively).

**Table 2:** Cox univariate and multivariate analysis for cardiovascular (a) and all-cause (b) mortality on the basis of eGFR strata.

	**eGFR** **>** **90**						**eGFR 60–90**						**eGFR** **<** **60**					
	***HR***	***95% CI***	***p***	***HR***	***95% CI***	***p***	***HR***	***95% CI***	***p***	***HR***	***95% CI***	***p***	***HR***	***95% CI***	***p***	***HR***	***95% CI***	***p***
**(A) CARDIOVASCULAR MORTALITY**																		
**Age, years**				1.10	1.08–1.13	<0.001				1.11	1.11–1.12	<0.001				1.07	1.06–1.09	<0.001
**Gender, male**				1.64	1.13–2.28	0.009				1.04	0.91–1.19	0.539				1.51	1.23–1.85	<0.001
**Uric acid, mg/dl**	1.47	1.31–1.80	<0.001	1.21	1.08–1.36	0.001	1.13	1.08–1.19	<0.001	1.13	1.07–1.19	<0.001	1.10	1.03 −1.17	0.002	1.05	0.99–1.12	0.119
**eGFR, ml/min per 1,73 m** ^**2**^				0.97	0.94–1.01	0.150				0.99	0.99–1.00	0.094				0.98	0.97–0.99	<0.001
**Diabetes, presence of**				2.83	1.92–4.15	<0.001				1.84	1.59–2.14	<0.001				2.04	1.67–2.50	<0.001
**Hypertension, presence of**				1.29	0.88–1.89	0.199				1.12	0.95–1.32	0.179				1.94	1.47–2.57	<0.001
**(B) ALL-CAUSE MORTALITY**																		
**Age, years**				1.10	1.09–1.11	<0.001				1.10	1.09–1.10	<0.001				1.07	1.06–1.08	<0.001
**Gender, male**				1.37	1.10–1.70	0.004				1.15	1.05–1.27	0.004				1.63	1.41–1.90	<0.001
**Uric acid, mg/dl**	1.22	1.14–1.30	<0.001	1.15	1.07–1.23	<0.001	1.11	1.08–1.15	<0.001	1.11	1.07–1.47	<0.001	1.10	1.05–1.15	<0.001	1.05	1.00–1.10	0.050
**eGFR, ml/min per 1,73 m** ^**2**^				0.99	0.97–1.01	0.409				1.00	0.99–1.00	0.280				0.98	0.98–0.99	<0.001
**Diabetes, presence of**				1.93	1.49–2.49	<0.001				1.65	1.48–1.85	<0.001				1.99	1.71–2.32	<0.001
**Hypertension, presence of**				1.06	0.85–1.32	0.588				1.14	1.01–1.28	0.028				1.59	1.31–1.93	<0.001

*HR, hazard ratio; CI, confidence interval; ACM, all-cause mortality; CVM, cardiovascular mortality; eGFR, estimated glomerular filtration rate (ml/min/1.73 m^2^)*.

On the contrary, independent eGFR predictive role on mortality did not seem to change on the basis of the presence or absence of HU based on URRAH cut off [adjusted HR for CVM 0.99 CI 95% [0.98–0.99], *p* < 0.0001 and 0.99 CI 95% [0.99–0.99] *p* < 0.0001 in individuals with and without HU respectively; HR for ACM 0.99 CI 95% [0.99–0.99], *p* < 0.0001 and 0.99 CI 95% [0.99–0.99] *p* = 0.0149 in individuals with and without HU, respectively, data not shown].

### Search for eGFR-Specific Cut Off Values of SUA

ROC curve analysis yielded plausible eGFR stage specific cut-off values of SUA for CVM (cut off points of SUA = 4,1 – 5,8 and 6,9 mg/dL in subjects with GFR >90, 60–90 and <60 ml/min/1.73 m^2^, respectively), and ACM (cut off points of SUA = 5,1 – 4,8 and 6,8 mg/dL in subjects with GFR >90, 60–90 and <60 ml/min/1.73 m^2^, respectively). These are summarized along with ROC curves parameters in Supplementary Table 3.

**Figure 2:**
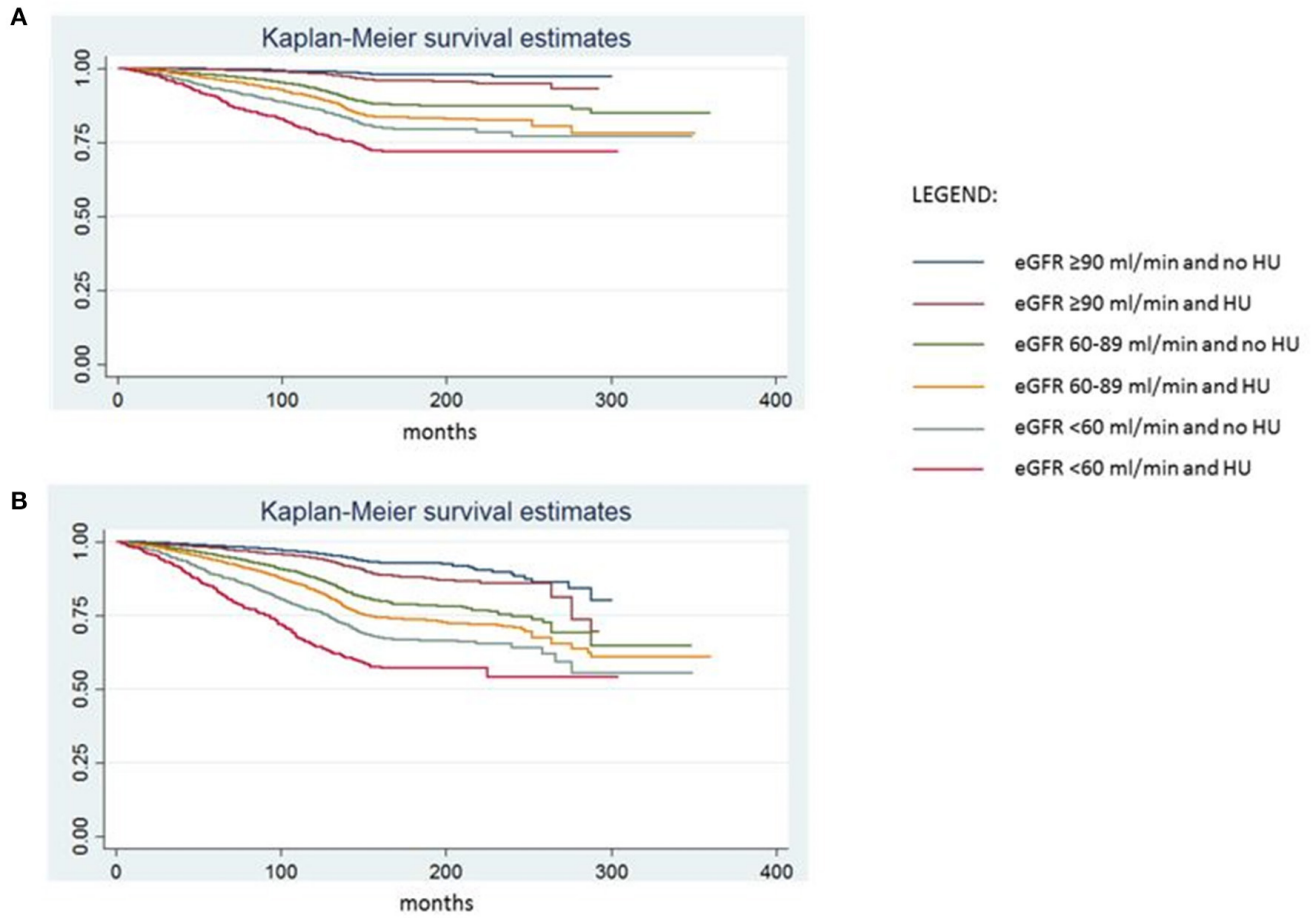
Survival without cardiovascular **(A)** and all-cause mortality **(B)** on the basis of eGFR strata and presence/absence of hyperuricemia.

Furthermore, we performed Kaplan-Meier survival curves analysis, in all participants on the basis of different eGFR stages (>90, 60–90 and <60 ml/min/1.73 m^2^). For each eGFR strata the additive presence of CKD specific definition of HU at baseline significantly decreased the survival proportion both for CV and ACM (Log Rank test *p* < 0.0001; Figure 2).

### Uric Acid According With eGFR Strata as Predictor of Outcomes

Supplementary Table 4 shows the unadjusted predictive role for CVM and ACM of all the available variables. In the multivariate analysis the risk of CVM and ACM increased by 10–20% in the different models, for each increase of 1 mg/dl of SUA, even after including eGFR strata and albuminuria as covariates (Table 3).

**Table 3:** Cox Multivariate Analysis for cardiovascular (a) and all-cause (b) mortality.

	**Multivariate model 1**			**Multivariate model 1b**			**Multivariate model 2**			**Multivariate model 3**		
	**HR**	**95% CI**	***p***	**HR**	**95% CI**	***p***	**HR**	**95% CI**	***p***	**HR**	**95% CI**	***p***
**(A) RISK FACTORS for CARDIOVASCULAR MORTALITY**												
**Age, years**	1.11	1.11–1.12	<0.001	1.11	1.10–1.11	<0.001	1.10	1.10–1.11	<0.001	1.10	1.09–1.12	<0.001
**Gender, male**	1.09	0.98–1.20	0.104	1.15	1.04–1.28	0.008	1.16	1.04–1.29	0.006	1.13	0.85–1.54	0.418
**Uric acid, mg/dl**	1.14	1.11–1.18	<0.001	1.12	1.08–1.16	<0.001	1.12	1.08–1.16	<0.001	1.21	1.09–1.34	<0.001
**eGFR, ml/min per 1.73 m** ^**2**^				0.99	0.99–0.99	0.002						
**eGFR**, **>90 ml/min per 1.73 m**^**2**^							Ref					
**eGFR 90 – 60 ml/min per 1.73 m** ^**2**^							1.30	1.08–1.56	0.005			
**eGFR** ** <60 ml/min per 1.73 m**^**2**^							1.33	1.07–1.65	0.009			
**Diabetes, presence of**							1.99	1.77–2.23	<0.001	1.97	1.37–2.84	<0.001
**Hypertension, presence of**							1.33	1.17–1.52	<0.001	3.56	1.92–6.60	<0.001
**Albuminuria, presence of**										2.47	1.69–3.60	<0.001
**(B) RISK FACTORS for ALL-CAUSE MORTALITY**												
**Age, years**	1.10	1.09–1.10	<0.001	1.10	1.09–1.10	<0.001	1.09	1.09–1.10	<0.001	1.08	1.07–1.09	<0.001
**Gender, male**	1.21	1.13–1.30	<0.001	1.24	1.15–1.34	<0.001	1.26	1.17–1.35	<0.001	1.01	0.82–1.25	0.901
**Uric acid, mg/dl**	1.11	1.09–1.14	<0.001	1.10	1.07–1.13	<0.001	1.10	1.07–1.13	<0.001	1.10	1.02–1.18	0.015
**eGFR, ml/min per 1.73 m** ^**2**^				0.99	0.99–1.00	0.087						
**eGFR**, **>90 ml/min per 1.73 m**^**2**^							Ref					
**eGFR 90 – 60 ml/min per 1.73 m** ^**2**^							1.02	0.91–1.14	0.762			
**eGFR** ** <60 ml/min per 1.73 m**^**2**^							1.04	0.90–1.19	0.600			
**Diabetes, presence of**							1.78	1.64–1.94	<0.001	1.99	1.52–2.59	<0.001
**Hypertension, presence of**							1.24	1.13–1.35	<0.001	5.60	3.38–9.28	<0.001
**Albuminuria, presence of**										2.11	1.60–2.78	<0.001

*HR, hazard ratio; CI, confidence interval; ACM, all-cause mortality; CVM, cardiovascular mortality; eGFR, estimated glomerular filtration rate; LDL, low-density lipoprotein*.

**Figure 3:**
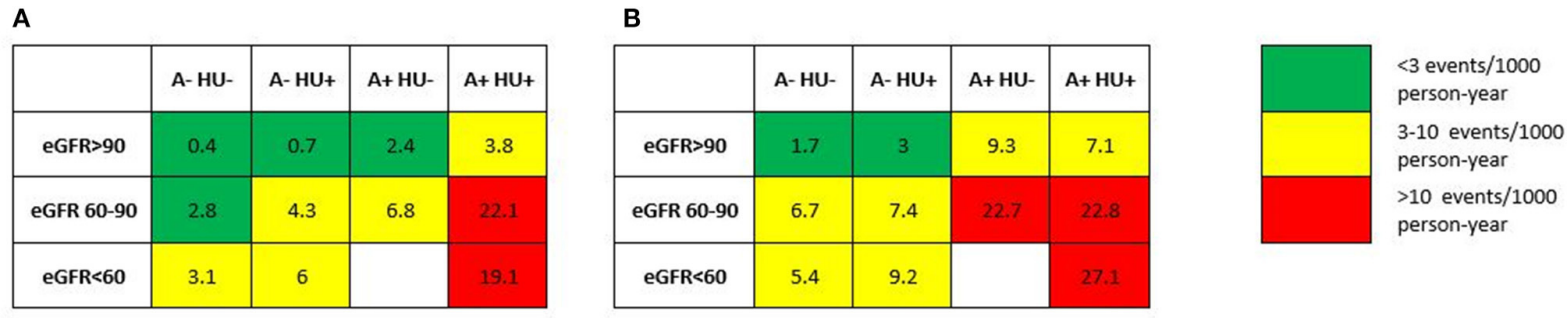
Cumulative incidence of cardiovascular **(A)** and all-cause mortality **(B)** on the basis of eGFR strata and presence/absence of hyperuricemia. A−, Normoalbuminuria; A+, Albuminuria; HU−, no hyperuricemia; HU+, hyperuricemia specific for eGFR strata and outcome.

During 58,181 person-years of follow-up, cumulative rate of CVM in patients with eGFR <90, 60–90 and <60 ml/min/1.73 m^2^, each one without and with albuminuria, was 2.1, 8.3, 7.1, 22.8, 6.3 and 27.1 per 1,000 person-years, respectively (Supplementary Table 1). The relationship between eGFR strata, albuminuria and CKD-specific HU is presented in Figure 3: patients with a combination of lower eGFR strata, albuminuria and/ or HU, showed the higher cumulative CVM and ACM rate.

## Discussion

In a large cohort of subjects at high cardiovascular risk from the URRAH database in Italy, baseline SUA values as well as the presence of CKD and each one of its components were potent, independent predictors of future CVM and ACM over a median follow-up of 9.8 years.

Hyperuricemia has previously been shown to be related to increased CVM and ACM in a variety of different clinical conditions such as hypertension (12), diabetes (16), chronic heart failure (17), as well as in the general population (18–20). Less is known about the association of increased SUA with outcomes in patients with CKD (7, 8, 21, 22) and whether HU is simply a marker of lower eGFR or it is causally associated with adverse outcomes in CKD.

Chronic kidney disease and each one of its components, namely reduced eGFR and increased albuminuria, have been traditionally regarded as powerful predictors of unfavorable outcome, especially CV morbidity and mortality (3, 23, 24). In the present study, we found that progressively lower values of eGFR are associated to the risk of CVM as well as ACM (Figure 1A). The same holds true for quartiles of SUA (Figure 1B).

GFR is unquestionably one of the main determinants of SUA levels, thus HU is a typical finding in patients with CKD although the independent pathogenetic contribution of each one of these variables is at present unclear. As a matter of fact, the relationship between HU and renal damage is thought to be bidirectional with one variable possibly contributing to influence the other one. The URRAH database with its large baseline cohort and relatively long-term follow-up, provided an ideal setting to investigate this interaction between SUA levels and eGFR strata in determining death. Results of interaction effect regression analysis indicate that SUA and eGFR interplay in determining mortality and, for the first time to our knowledge, we described as the independent predictive power of SUA tends to decrease along with the severity of renal impairment (Table 2).

Previous studies conducted on the URRAH database (12) have identified operational cut-offs for UA (i.e., 5.6 and 4.7 mg/dl) in terms of CVM and ACM in a high-risk general cohort from multiple sites in Italy. Nevertheless, it is at present unclear which threshold values should be applied to define asymptomatic HU in the presence of CKD, in view of the inverse relationship between eGFR and the prevalence of hyperuricemia (25). We looked at identifying specific values of SUA with predictive power for CVM and ACM in this setting. Our ROC curve analysis indicates that SUA values of 4.8 and 6.8 respectively could be valuable threshold to predict future ACM in patients with low eGFR values, namely between 60 and 90 and below 60 ml/min. Similarly, we found that a SUA of 5.8 and 6.9 mg/dl may be used to predict CVM in the presence of progressively lower eGFR values (Supplementary Table 3). Altogether, our data suggest that in the context of greater global risk, as it is the case when GFR is even slightly reduced, SUA becomes a significant correlate of unfavorable outcome only at serum concentration greater than what is observed in subjects with normal renal function. By a pathophysiological point of view, these data are consistent with the hypothesis that while HU may simply result from reduced kidney clearance, uric acid perpetuates glomerular injury, leading to a progressive vicious cycle of further renal damage and thereafter to an increased CV risk. The mechanisms deemed to be implicated in the progression of SUA mediated renal damage are likely multiple and include cytokine release, promotion of endothelial, vascular, and interstitial damage, upregulation of the renin–angiotensin aldosterone system, and changes in glomerular hemodynamics leading to glomerulosclerosis and fibrosis (26).

Our findings on specific SUA levels predicting CVM and ACM in different CKD strata may also provide an explanation for the relatively conflicting data previously reported in the literature on the relationship between HU and risk in the presence of CKD (2, 27). In fact, renal function may have acted as a confounder in the relationship between HU and CVR when data were analyzed in aggregate without adjusting for GFR values.

Aside from a meta-analysis of 11 cohort studies including 11,050 participants, which has previously shown an association between elevated SUA levels and increased risk of CVM (28) in renal patients, this is, to our knowledge, the largest report identifying specific predictive SUA values for CVM in the presence of CKD. As a matter of fact, KM analysis showed that HU has an additive prognostic power on top of each progressively strata of eGFR in predicting both CVM and ACM (Figure 2).

Several potential mechanisms support the pathogenic link between higher SUA levels and higher CVM. Hyperuricemia is closely related to metabolic syndrome, obesity and diabetes, hypertension, and cardiovascular events which are common risk factors for CKD and mortality (29–34). Several experimental studies defined potential pathways linking UA to CV lesions. The mechanisms include, inflammation, oxidative stress, activation of the renin-angiotensin aldosterone system (RAAS), endothelial dysfunction, and proliferation of vascular smooth muscle cells (VSMC), resulting in increased CV risk (35, 36).

HU and each one of the two measures of CKD, reduced eGFR and increased albuminuria, all retain an independent predictive role to the incidence of CVM and ACM as indicated by results of Cox multivariate analysis (Table 3). Moreover, patients with CKD stage 3a according to NKF classification, i.e., an eGFR lower than 60 ml/min, show a 6-fold greater incidence of fatal cardiovascular events over a median follow up time of 9.8 years as compared to those with a preserved renal function, without albuminuria or HU. This figure doubles in the presence of HU and increases in a dramatic fashion up to an almost 40 times greater value when an increased albuminuria is also concomitant (Figure 3).

Thus, our study indicates that both CKD and HU have independent role in determining CVM and ACM. HU, retains an additive predictive power on top of reduced GFR strata, although at a slightly higher threshold value as compared to what has been reported in high risk, unselected cohorts.

Our study has some limitations as well as several strengths that should be mentioned. Among the first ones, we must acknowledge that laboratory parameters, including serum creatinine and SUA were not measured in a single, centralized laboratory. Moreover, time-varying changes in UA levels during the follow-up period were not available. Furthermore, our data may not be applicable to the population at high CV risk at large, as the vast majority of participants were of white origin. Moreover, because this was an observational study, only associations, but no cause-effect relationships, can be inferred. Finally, we did not have information on renal progression or the need of kidney replacement treatment, which may affect CV risk factors changes overtime. On the other hand, the large size and the fact that all strata of eGFR are widely represented as well as the representative geographical distribution of the recruiting centers (in Italy) and the relatively long follow-up period, do contribute to make our results a reliable representation of real-life clinical condition.

In conclusion, HU is a risk factor for CVM and ACM additively to eGFR strata and albuminuria in patients at CV risk. These data suggest that although CKD is a major determinant of the presence and degree of HU, these two conditions may have, at least in part, different pathogenetic mechanisms by which they both contribute to the excess of CV morbidity and mortality. Moreover, a higher threshold values (≥7 mg/dl) should be applied to define asymptomatic HU in the presence of CKD.

## Data Availability Statement

The raw data supporting the conclusions of this article will be made available by the authors, without undue reservation.

## Ethics Statement

The studies involving human participants were reviewed and approved by the Ethical Committee of the coordinating center at the Division of Internal Medicine of the University of Bologna (no. 77/2018/Oss/AOUBo). The patients/participants provided their written informed consent to participate in this study.

## Author Contributions

CB, GG, RP, MC, FV, ER, AV, MM, and CF: research idea and study design. ER, FV, RP, MC, and GL: data analysis/interpretation. ER, FV, and GL: statistical analysis. CB, GG, RP, MC, FV, ER, AV, MM, CF, and GP: supervision or mentorship. Each author contributed important intellectual content during manuscript drafting or revision, accepts personal accountability for the author's own contributions, and agrees to ensure that questions pertaining to the accuracy or integrity of any portion of the work are appropriately investigated and resolved. All authors contributed to the article and approved the submitted version.

## Funding

This work has been conducted with an unrestricted grant from the Fondazione of the Italian Society of Hypertension (Grant Number: MIOL). The funders had no role in study design, data collection and analysis, decision to publish, or preparation of the manuscript.

## Conflict of Interest

The authors declare that the research was conducted in the absence of any commercial or financial relationships that could be construed as a potential conflict of interest.

## Publisher's Note

All claims expressed in this article are solely those of the authors and do not necessarily represent those of their affiliated organizations, or those of the publisher, the editors and the reviewers. Any product that may be evaluated in this article, or claim that may be made by its manufacturer, is not guaranteed or endorsed by the publisher.
