# Supplementary material for: Serum Uric Acid and Kidney Disease Measures Independently Predict Cardiovascular and Total Mortality: The Uric Acid Right for Heart Health (URRAH) Project
Source: Front Cardiovasc Med. 2021 Sep 27;8:713652. doi: 10.3389/fcvm.2021.713652 (PMC8502977; doi:10.3389/fcvm.2021.713652)
Supplement: Supplementary file 1 [file Data_Sheet_1.pdf]

Supplemental Table 1. Baseline clinical characteristics of study patients on the basis of glomerular filtration rate and albuminuria.

	All	eGFR>90 without albuminuria	eGFR >90 with albuminuria	eGFR 60-90 without albuminuria	eGFR 60-90 with albuminuria	eGFR<60 without albuminuria	eGFR<60 with albuminuria	P value
N	5170	1713	138	2240	234	692	93	
Age, years	58 ±15	48.1 ±12.9	52.3 ±13.6	60.2 ±12.9	65.3 ±12.4	66.8 ±12.8	71.4 ±10.2	<0.0001
Male sex, %	54.0	69.2	69.2	54.1	52.6	14.6	35.5	<0.0001
Body mass index, kg/m^2	26.7 ±4.3	25.6 ±4.2	27.2 ±5.2	26.5 ±4.0	27.5 ±4.1	27.4 ±4.1	28.3 ±4.5	<0.0001
Smokers, %	23.6	22.9	30.5	21.5	20.3	31.6	23.1	<0.0001
Hypertension, %	67.8	60.2	72.9	72.9	83.6	61.4	80.7	<0.0001
Systolic blood pressure, mmHg	143.7 ±23.5	142.4 ±19.6	144.7 ±22.0	150.4 ±20.9	150.4 ±21.3	146.7 ±21.4	151.1 ±28.2	<0.0001
Diastolic blood pressure, mmHg	85.3 ±12.7	86.1 ±13.9	88.2 ±13.3	88.0 ±13.2	89.0 ±12.6	79.8 ±12.0	83.1 ±14.1	<0.0001
Heart rate, bpm	72 ±12	69 ±11	71 ±12	69 ±11	71 ±11	64 ±11	6 ±12	<0.0001
Creatinine, mg/dl	0.93 ±0.26	0.79 ±0.13	0.75 ±0.12	0.97 ±0.14	0.93 ±0.14	1.20 ±0.17	1.39 ±0.45	<0.0001
eGFR, ml/min per 1.73m^2	81.5 ±20.0	103.2 ±9.9	102.7 ±9.4	76.2 ±8.5	76.2 ±9.0	50.7 ±7.3	46.4 ±10.4	<0.0001
eGFR<60 ml/min per 1.73m^2, %	15.2	0	0	0	0	100	100	<0.0001
Serum uric acid, mg/dl	5.03 ±1.42	4.70 ±1.27	5.03 ±1.45	5.18 ±1.27	5.49 ±1.42	6.04 ±1.30	6.37 ±1.25	<0.0001
Hyperuricemia according to URRAH cut-off for CVM, %	34.3	21.2	32.3	34.1	44.0	59.5	69.9	<0.0001
Hyperuricemia according to eGFR strata specific cut-off for CVM, %	42.0	64.8	68.7	28.3	38.0	24.1	37.6	<0.0001
Hyperuricemia according to URRAH cut-off for ACM, %	59.5	44.7	54.0	61.4	66.7	84.5	92.5	<0.0001
Hyperuricemia according to eGFR strata specific cut-off for ACM, %	44.6	32.9	42.9	58.3	63.7	24.1	37.6	<0.0001
Gout, %	0.3	0.1	0	0.2	0.5	0.4	2.5	0.0041
Allopurinol use, %	0.8	0.5	2.4	0.3	1.0	1.7	7.7	<0.0001
Hemoglobin, g/dl	14.4 ±1.4	14.5 ±1.3	14.3 ±1.4	14.5 ±1.3	14.2 ±1.4	14.3 ±1.6	13.7 ±1.3	0.0001
Glicemia, mg/dl	99 ±25	90 ±14	97 ±30	95 ±16	102 ±27	101 ±23	110 ±37	<0.0001
Diabetes, %	6.6	1.6	9.7	6.7	22.0	9.2	30.4	<0.0001
Total cholesterol, mg/dl	213 ±39	201 ±38	198 ±42	213 ±37	208 ±37	211 ±39	195 ±44	<0.0001
HDL-cholesterol, mg/dl	55 ±19	55 ±14	53 ±16	52 ±15	50 ±13	48 ±14	47 ±13	<0.0001

**LDL-cholesterol, mg/dl**	133 ±38	126 ±36	120 ±36	138 ±35	134 ±35	137 ±35	119 ±43	<0.0001
**Triglycerides, mg/dl**	129 ±79	106 ±66	121 ±86	121 ±70	128 ±63	133 ±73	145 ±64	<0.0001
**ACE inhibitors,%**	15.8	10.1	19.6	16.0	26.5	20.6	30.8	<0.0001
**Angiotensin II receptor blockers, %**	7.5	4.3	16.4	5.6	15.1	12.2	23.9	<0.0001
**Calcium channel blockers,%**	5.9	2.6	11.9	5.7	14.2	7.9	23.1	<0.0001
**Beta blockers, %**	9.9	7.4	21.2	8.4	16.1	12.1	24.7	<0.0001
**Diuretics, %**	13.4	5.7	17.5	13.0	23.3	23.8	31.2	<0.0001
**Statins, %**	10.7	5.8	19.3	8.6	16.2	18.9	34.8	<0.0001

Data presented as mean ± standard deviation or percentage.

Abbreviations: eGFR, estimated glomerular filtration rate (ml/min per 1.73 m^2); ACM, all-cause mortality; CVM, cardiovascular mortality; SUA, serum uric acid; HDL, high-density lipoprotein; LDL, low-density lipoprotein; ACE, angiotensin converting enzyme.

Supplemental Table 2. Baseline clinical characteristics of study patients on the basis of all-cause and cardiovascular outcomes.

	No CVD death	CVD death	p	No all-cause death	All-cause death	P value
N	20,376	1,582		18,833	3,130	
Age, years	56.8 ±14.6	72.9 ±9.4	<0.0001	55.7 ±14.3	71.4 ±10.1	<0.0001
Male sex, %	49.8	46.5	0.0112	49.5	49.8	0.7605
Body mass index, kg/m^2	26.7 ±4.3	26.8 ±4.6	0.1804	26.7 ±4.3	26.7 ±4.5	0.6492
Smokers, %	24	17.2	<0.0001	24.1	20.2	<0.0001
Hypertension, %	61.1	73.5	<0.0001	60.3	72.3	<0.0001
Systolic blood pressure, mmHg	142.6 ±23.0	158.0 ±25.5	<0.0001	141.7 ±22.6	156.0 ±25.2	<0.0001
Diastolic blood pressure, mmHg	85.2 ±12.6	86.0 ±13.3	0.0216	85.1 ±12.6	86.3 ±12.9	<0.0001
Heart rate, bpm	71 ±12	75 ±12	<0.0001	71.2 ±12.0	74.6 ±11.9	<0.0001
Creatinine, mg/dl	0.93 ±0.25	1.01 ±0.33	<0.0001	0.93 ±0.25	1.00 ±0.31	<0.0001
eGFR, ml/min per 1.73m^2	82.5 ±19.6	68.4 ±17.1	<0.0001	83.3 ±19.5	70.8 ±17.6	<0.0001
Chronic kidney disease, %	16.2	28.2	<0.0001	16.1	22.8	<0.0001
eGFR<60 ml/min per 1.73m^2, %	13.2	30	<0.0001	12.4	26.5	<0.0001
Normo-micro-macroalbuminuria, %	90.2 - 8.9 - 0.9	78.6 - 20.8 - 0.6	<0.0001	90.4 - 8.7 - 0.9	82.0 - 16.9 - 1.1	<0.0001
Serum uric acid, mg/dl	4.90 ±1.41	5.96 ±1.51	<0.0001	4.96 ±1.40	5.42 ±1.49	<0.0001
Hyperuricemia according to URRAH cut-off for TM, %	54.3	67.5	<0.0001	53.4	66.3	<0.0001
Hyperuricemia according to URRAH cut-off for CVM, %	47.7	52.2	0.0009	28.9	40.1	<0.0001
Gout, %	1	2.1	0.0001	0.9	2	<0.0001
Allopurinol use, %	1.4	2.1	0.046	1.4	0.089	<0.0001
Hemoglobin, g/dl	14.4 ±1.4	14.4 ±1.5	0.4295	14.4 ±1.4	14.4 ±1.5	0.4368
Glucose, mg/dl	97.7 ±23.3	114.5 ±41.4	<0.0001	96.8 ±22.0	110.4 ±37.0	<0.0001
Diabetes, %	9.7	26.1	<0.0001	8.8	23.1	<0.0001
Cholesterol, mg/dl	212.6 ±39.4	213.7 ±39.7	0.2896	212.8 ±39.2	212.0 ±40.7	0.346
HDL-cholesterol, mg/dl	54.8 ±18.9	53.1 ±16.2	0.0009	55.0 ±19.1	52.9 ±16.0	<0.0001
LDL-cholesterol, mg/dl	133.0 ±38.0	133.9 ±35.8	0.3969	133.1 ±38.1	132.8 ±36.6	0.7064
Triglycerides, mg/dl	128.3 ±78.9	138.9 ±77.5	<0.0001	127.9 ±78.7	135.9 ±79.4	<0.0001
ACE inhibitors,%	8.3	10.6	0.0015	8.4	9.4	0.062

**Angiotensin II receptor blockers, %**	9.4	4.7	<0.0001	9.8	4.6	<0.0001
**Calcium channel blockers,%**	16.5	15.6	0.3406	16.9	13.9	<0.0001
**Beta blockers, %**	5.6	1.9	<0.0001	6	1.6	<0.0001

Data presented as mean ± standard deviation or percentage.

Abbreviations: eGFR, estimated glomerular filtration rate; ACM, all-cause mortality; CVM, cardiovascular mortality; SUA, serum uric acid; HDL, high-density lipoprotein; LDL, low-density lipoprotein; ACE, angiotensin converting enzyme.

Supplemental Table 3. Survival ROC curve identifying the best threshold of serum uric acid for cardiovascular and all-cause mortality according to eGFR strata.

eGFR <60 ml/min per 1.73m^2

Outcomes	*Cut-off Points, mg/dl*	*Std Err*	*AUC*	*95% CI*	*Sensitivity*	*Specificity*
Cardiovascular mortality	6.9	0.015	0.5398	0.5224 - 0.5574	0.30	0.77
All-cause mortality	6.8	0.012	0.5406	0.5230 - 0.5580	0.29	0.78

eGFR 60-90 ml/min per 1.73m^2

Outcomes	*Cut-off Points, mg/dl*	*Std Err*	*AUC*	*95% CI*	*Sensitivity*	*Specificity*
Cardiovascular mortality	5.8	0.0099	0.5409	0.5316 - 0.5501	0.36	0.71
All-cause mortality	4.8	0.0073	0.5379	0.5286 - 0.5472	0.64	0.43

eGFR >90 ml/min per 1.73m^2

Outcomes	*Cut-off Points, mg/dl*	*Std Err*	*AUC*	*95% CI*	*Sensitivity*	*Specificity*
Cardiovascular mortality	4.1	0.0224	0.6079	0.5968 - 0.6190	0.79	0.39
All-cause mortality	5.1	0.0141	0.5806	0.5693 - 0.5917	0.42	0.69

Abbreviations: eGFR, estimated glomerular filtration rate.

Supplemental Table 4. Unadjusted risk factors for cardiovascular and all-cause mortality

	RISK FACTORS for CVM			*RISK FACTORS for ACM*		
	HR	***95% CI***	***p***	***HR***	***95% CI***	***p***
**Age, years**	1.11	1.11 - 1.12	**<0.0001**	1.10	1.09 - 1.10	**<0.0001**
**Male sex**	0.93	0.84 - 1.02	0.1349	1.05	0.98 - 1.13	0.1657
**Serum uric acid, mg/dl**	1.27	1.23 - 1.32	**<0.0001**	1.23	1.20 - 1.26	**<0.0001**
**eGFR, ml/min per 1.73m²**	0.96	0.96 - 0.97	**<0.0001**	0.97	0.97 - 0.97	**<0.0001**
**eGFR, >90 ml/min per 1.73m²**	Ref			Ref		
**eGFR 90 – 60 ml/min per 1.73m²**	4.47	3.76 - 5.31	**<0.0001**	1.11	1.08 - 1.15	**<0.0001**
**eGFR <60 ml/min per 1.73m²**	8.52	7.09 - 10.24	**<0.0001**	1.11	1.06 - 1.15	**<0.0001**
**Diabetes, presence of**	3.55	3.17 - 3.98	**<0.0001**	3.03	2.79 - 3.30	**<0.0001**
**Hypertension, presence of**	2.97	2.61 - 3.39	**<0.0001**	1.87	1.45 - 2.40	**<0.0001**
**Albuminuria, presence of**	4.37	3.01 - 6.32	**<0.0001**	3.43	2.61 - 4.51	**<0.0001**
**Smoker, %**	1.60	1.44 - 1.78	**<0.0001**	0.71	0.66 - 0.76	**<0.0001**
**LDL-cholesterol, mg/dl**	0.99	0.99 - 0.99	**0.0008**	0.99	0.99 - 0.99	**<0.0001**
**Body mass index, Kg/m²**	1.01	1.00 - 1.02	0.0585	1.00	0.99 - 1.01	**0.1847**
**Systolic blood pressure, mmHg**	1.02	1.02 - 1.02	**<0.0001**	1.02	1.02 - 1.02	**<0.0001**
**CKD, presence of**	1.81	1.62 - 2.02	**<0.0001**	1.37	1.26 - 1.49	**<0.0001**
**Hyperuricemia, according to URRAH cut offs**	1.85	1.67 - 2.04	**<0.0001**	1.67	1.55 - 1.80	**<0.0001**
**Total cholesterol, mg/dl**	0.99	0.99 - 1.00	0.2271	0.99	0.99 – 1.00	**<0.0001**
**Triglycerides, mg/dl**	1.00	1.00 - 1.00	**<0.0001**	1.00	1.00 - 1.00	**<0.0001**

Abbreviations: HR, hazard ratio; CI, confidence interval; ACM, all-cause mortality; CVM, cardiovascular mortality; eGFR, estimated glomerular filtration rate; LDL, low-density lipoprotein; CKD, chronic kidney disease.
